# Medullary Carcinoma of the Ascending Colon in an Older Woman: A Case Report

**DOI:** 10.70352/scrj.cr.26-0022

**Published:** 2026-09-05

**Authors:** Ayana Kishimoto, Tetsu Yamamoto, Takahito Taniura, Kazunari Ishitobi, Keisuke Inoue, Shunsuke Kaji, Takayuki Tanaka, Takeshi Matsubara, Masaaki Hidaka

**Affiliations:** Department of Digestive and General Surgery, Shimane University Faculty of Medicine, 89-1 Enya-cho, Izumo, Shimane 693-8501, Japan

**Keywords:** medullary carcinoma, older, colon, laparoscopic surgery

## Abstract

**INTRODUCTION:**

Medullary carcinoma of the colon is a rare histological subtype of poorly differentiated or undifferentiated adenocarcinoma, with a reported incidence of 0.05%–0.08%. It typically affects older women, is more frequently located in the right colon, and is characterized by a high prevalence of mismatch repair deficiency, particularly microsatellite instability (MSI). In comparison to other poorly differentiated adenocarcinomas, medullary carcinoma generally exhibits a lower rate of lymph node or distant metastasis and carries a relatively favorable prognosis. Due to its recent classification and rarity, reported cases remain limited. We herein describe a case of surgically resected medullary carcinoma of the ascending colon in an older woman, along with a brief review of the relevant literature.

**CASE PRESENTATION:**

An 88-year-old woman presented with unintentional weight loss of 4 kg over 3 months. Colonoscopy revealed a type 2 tumor in the ascending colon, and biopsy demonstrated poorly differentiated adenocarcinoma. Imaging showed asymmetric wall thickening without lymph node or distant metastasis, and the clinical diagnosis was ascending colon cancer, cT3N0M0, Stage IIA. She underwent an elective laparoscopic right hemicolectomy with D3 lymphadenectomy. The postoperative course was uneventful. A histopathological examination showed sheets of atypical cells with enlarged nucleoli, expansive growth, a Crohn’s-like lymphoid reaction, and focal signet-ring-like features. Immunohistochemistry demonstrated positivity for MUC5AC, partial positivity for MUC2, focal positivity for CK20 and CDX-2, retained expression of MSH2 and MSH6, and loss of MLH1 and PMS2 expression, indicating deficient mismatch repair (dMMR), which supports the diagnosis of medullary carcinoma. The final pathological staging was pT3N0M0, Stage IIA. Given the absence of lymph node metastasis and the patient’s advanced age, adjuvant chemotherapy was not administered. She has remained recurrence-free for 4 years under active surveillance.

**CONCLUSIONS:**

Colonic medullary carcinoma is a rare tumor that predominantly occurs in older patients and generally demonstrates a more favorable prognosis than other poorly differentiated adenocarcinomas. Treatment decisions should be based primarily on tumor stage. Although preoperative diagnosis is often difficult, awareness of its characteristic features may help avoid excessive therapeutic de-escalation.

## INTRODUCTION

Colon cancers are predominantly well- or moderately differentiated adenocarcinomas, with poorly differentiated and undifferentiated variants occurring only infrequently.^1)^ Medullary carcinoma is classified as a subtype of poorly differentiated or undifferentiated adenocarcinoma, but generally has a more favorable prognosis than conventional poorly differentiated adenocarcinoma. It is exceedingly rare, with reported incidence rates of only 0.05%–0.08%.^2)^ It is characterized by its high incidence in middle-aged and older women, its relatively high incidence on the right side of the colon, and its relatively low incidence of lymph node and distant metastasis.^3)^ Approximately 80% of medullary carcinomas exhibit high microsatellite instability (MSI) due to mismatch repair deficiency, and these tumors generally have a more favorable prognosis than other poorly differentiated or undifferentiated adenocarcinomas.^4)^ Because of these characteristics, medullary carcinoma of the colon is a relatively new, classified histological subtype with fewer reported cases.

We herein describe a case of resected medullary colon cancer in an elderly patient, with a review of the relevant literature.

## CASE PRESENTATION

An 88-year-old woman presented to the outpatient department of a local hospital with a chief complaint of weight loss (4 kg over 3 months). Further examination revealed a type 2 tumor in the ascending colon on total colonoscopy. A histopathological examination demonstrated adenocarcinoma, and the patient was referred to our hospital for surgical treatment.

She had a history of a left femur fracture 5 months previously. She also had a 3-mm cerebral aneurysm and declined treatment.

Her height was 148 cm, and her body weight was 53 kg, with a BMI of 24.2 kg/m^2^. A physical examination revealed a flat, soft abdomen without a palpable mass.

Blood chemistry showed no anemia or inflammatory findings. Serum tumor markers revealed a carcinoembryonic antigen (CEA) level of 2.9 ng/mL (reference range, <5 ng/mL) and a carbohydrate antigen 19-9 (CA19-9) level of 44.7 U/mL (reference range, <37 U/mL).

At admission, the patient’s Barthel Index was 75/100 and her Clinical Frailty Scale score was 5/9, indicating mild dependence in ADL and mild frailty.

### Image findings

**Fig. 1:**
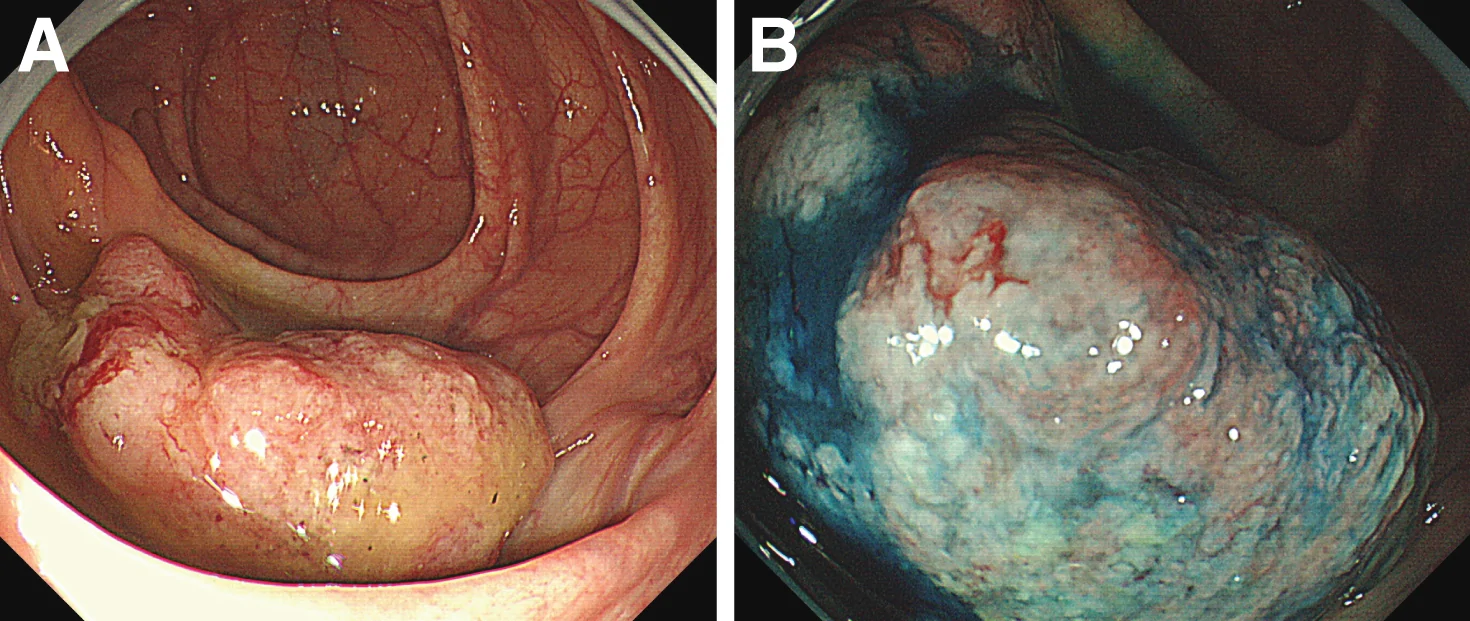
(**A**) Total colonoscopy showed a type 2 tumor in the ascending colon. The size of the tumor was 30 × 30 mm. (**B**) The glandular structures on the tumor surface were disorganized by the indigo carmine spray.

Total colonoscopy found a type 2 tumor in the ascending colon. Magnifying endoscopy and narrow-band imaging revealed a Vi high-grade pit pattern with a “rough margin” and “narrow lumen” on the tumor surface. In the indigo carmine-sprayed image, a superficially elevated component was observed surrounding the mass-like lesion. The glandular structures on the tumor surface showed architectural irregularity, size variation, and partial indistinctness, suggesting the possibility of muscularis propria invasion (**Fig. 1**). A tissue biopsy was performed.

**Fig. 2:**
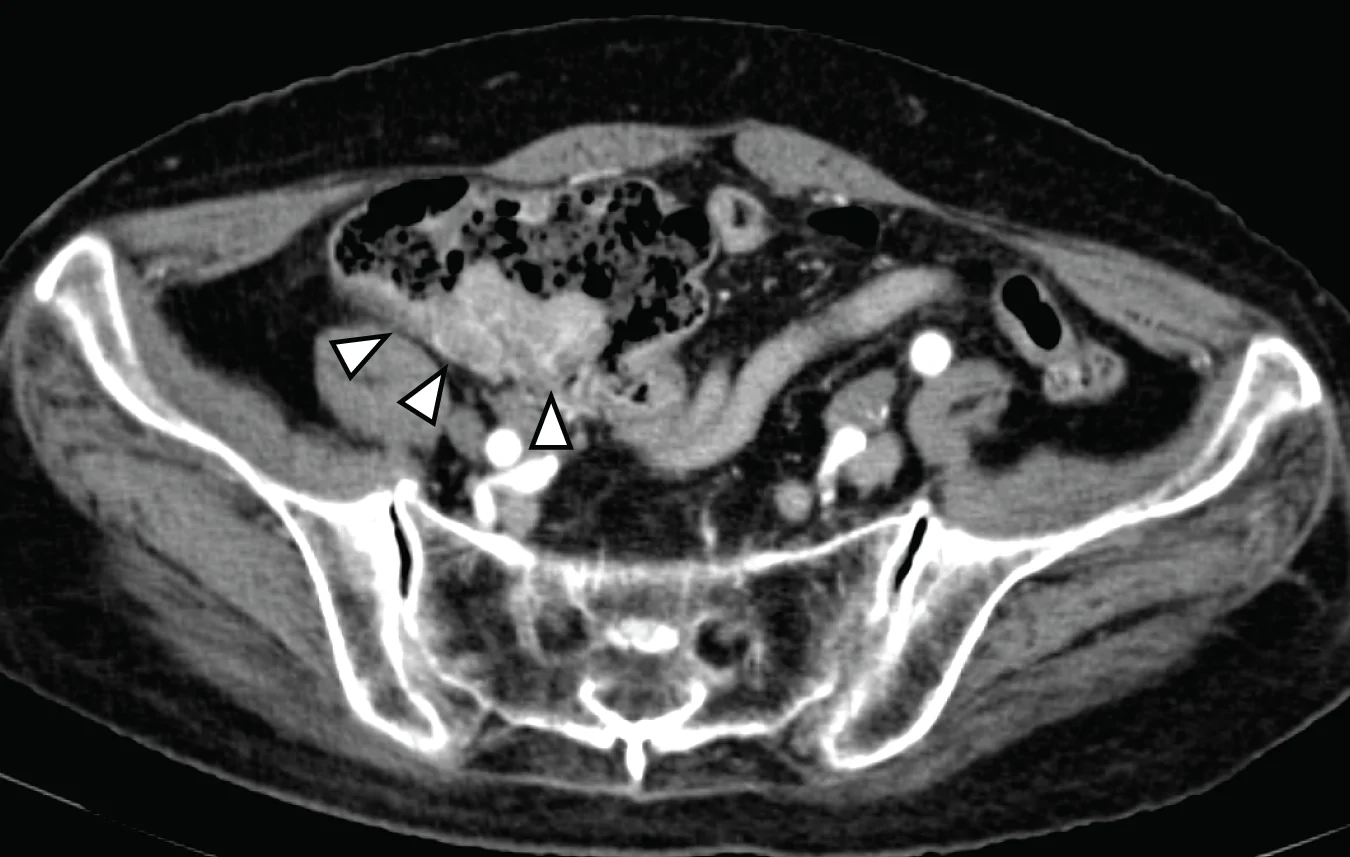
Abdominal contrast-enhanced CT showed wall thickening with contrast enhancement in the ascending colon (arrowhead). No lymph node enlargement was recognized.

A Gastrografin study revealed that the tumor measured approximately 3 cm. An examination of the biopsy specimen showed poorly differentiated adenocarcinoma. Abdominal contrast-enhanced CT scans showed asymmetric circumferential wall thickening of the ascending colon, but no lymph node swelling or distant metastasis (**Fig. 2**).

The patient was diagnosed with ascending colon cancer, cT3N0M0, cStage IIA, and was scheduled to undergo an elective laparoscopic right hemicolectomy.

### Operation findings

The patient was placed in the supine split-leg position under epidural and general anesthesia. First, the greater omentum was dissected, and then the omental bursa was exposed. The mesentery of the transverse colon was dissected from the lower edge of the pancreas. The incision of the transverse mesentery was extended to the hepatic flexure. The accessory right colic vein was recognized and dissected at the base (superior approach). After that, the white line was dissected, and the ascending colon was mobilized from the retroperitoneum. After recognition of the ileocolic vessels, lymph node dissection was performed around the base of these vessels. The ileocolic artery and vein were ligated separately at the junction with the superior mesenteric vein and were dissected. Then, lymph node dissection was performed along the anterior superior mesenteric vein. The right branch of the middle colic artery was ligated and dissected at the junction with the middle colic artery. The dissection line was determined approximately 10 cm from the tumor, and the mesentery of the right colon was dissected. The blood supply was confirmed using near-infrared radiation fluorescence imaging after the intravenous administration of ICG at a concentration of 0.2 mg/kg. The transverse colon and the ileum were dissected using an automatic suture device, and an intracorporeal overlap anastomosis was performed. The operation time was 295 min, and the estimated blood loss was less than 10 g. The patient’s postoperative course was uneventful, and she was discharged 33 days after surgery.

### Histological findings

**Fig. 3:**
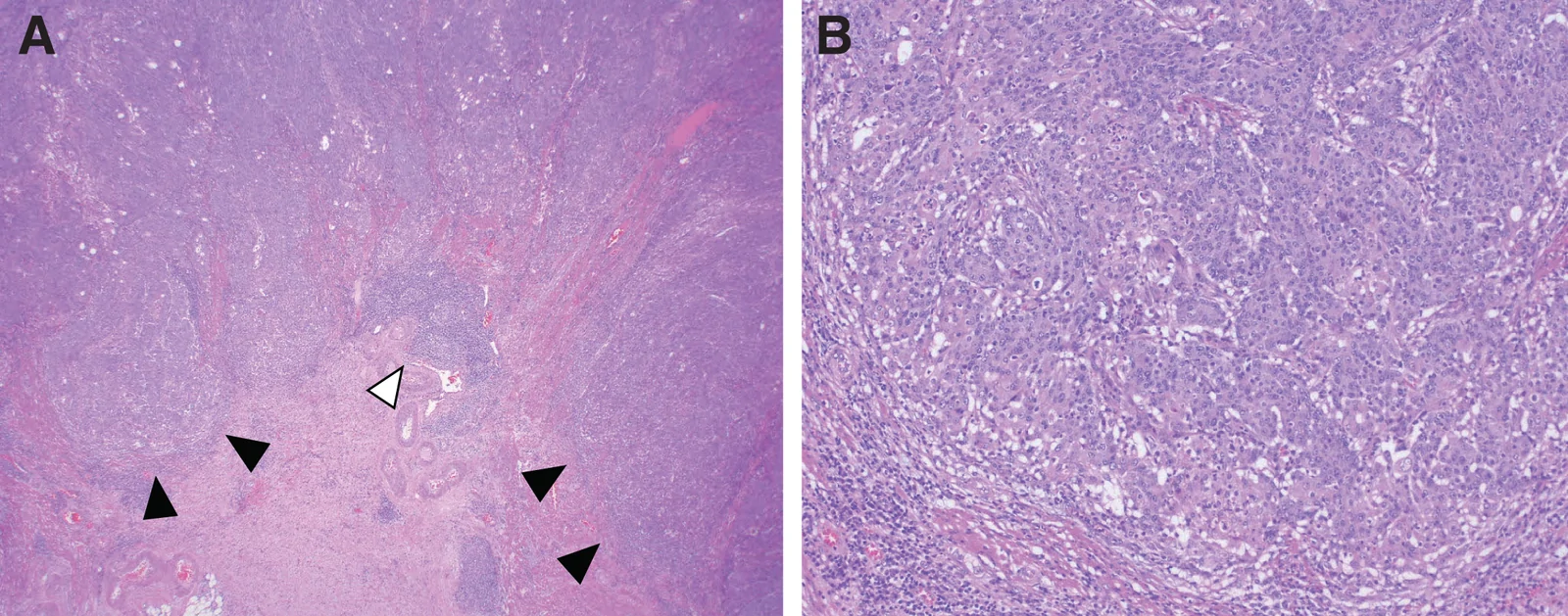
Hematoxylin and eosin staining showed (**A**) expansive proliferation (arrowhead), and Crohn’s-like lymphoid reaction in the gastrointestinal wall (white arrowhead), and (**B**) sheet-like clusters of atypical cells with distinct nucleoli.

An enlarged nucleolus and nuclear atypia were noted. The atypical cells formed sheet-like nests. In addition, expansive proliferation of the atypical cells and Crohn’s-like lymphoid reactions, characterized by aggregates of lymphocytes in the intestinal wall, were observed (**Fig. 3**). The coexistence of signet-ring-like tumor cells and adenocarcinoma was noted. Extracellular mucin production was also observed. The tumor consisted predominantly of medullary carcinoma (approximately 80%). Because the mucinous and signet ring cell-like components were difficult to distinguish histologically, they were evaluated together and accounted for approximately 15% of the tumor. The remaining 5% consisted of a conventional adenocarcinoma.

**Fig. 4:**
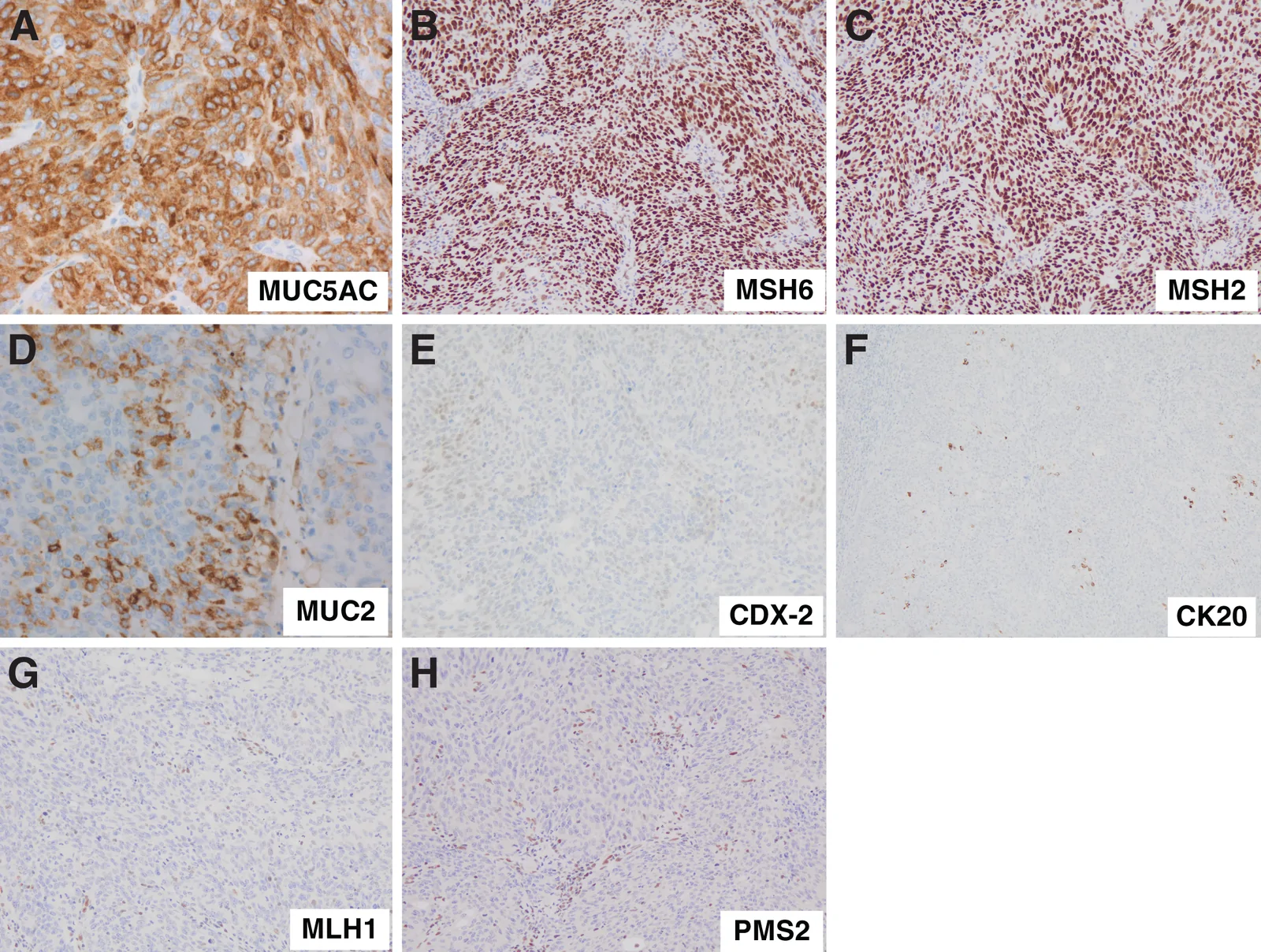
Immunohistochemical staining showed (**A**) positive staining for MUC5AC, (**B**) retained expression of MSH6, (**C**) retained expression of MSH2, (**D**) partial positivity for MUC2, (**E**) focal positivity for CDX-2, (**F**) focal positivity for CK20, (**G**) loss of MLH1 expression, and (**H**) loss of PMS2 expression in the tumor cells.

Immunohistochemical staining showed positivity for MUC5AC, partial positivity for MUC2, focal CK20 positivity, and focal CDX-2 positivity. Additional immunohistochemical staining for mismatch repair proteins demonstrated loss of MLH1 and PMS2 expression, with preserved expression of MSH2 and MSH6, indicating mismatch repair deficiency (dMMR) (**Fig. 4**). Taken together, these morphological and immunohistochemical findings supported the diagnosis of medullary carcinoma of the colon.

### Diagnosis and postoperative course

The final diagnosis was pT3N0M0, pStage IIA medullary carcinoma of the colon. It was decided that adjuvant chemotherapy would not provide additional benefit given the stage of the malignancy. Active surveillance was initiated, including 3-monthly evaluation of tumor markers (CEA and CA19-9), 6-monthly CT scans, and annual colonoscopy. No evidence of recurrence was observed 4 years postoperatively.

## DISCUSSION

Medullary carcinoma of the colon is a relatively new histological subtype of adenocarcinoma characterized by a solid growth pattern and poorly differentiated or undifferentiated cells.^5,6)^ It was previously classified as a subtype of the poorly differentiated or undifferentiated adenocarcinoma. Still, it has since been recognized as having a better prognosis among these histological categories.^7)^ The 1-year and 2-year overall survival (OS) rates of medullary carcinoma have been reported to be 92.7% and 73.8%, respectively, which are superior in comparison to those of poorly differentiated adenocarcinoma (1-year, 70.5%; 2-year, 58.4%).^4,5)^

Although the incidence of medullary carcinoma has been reported to be 0.05%–0.08%, the actual incidence is unclear because it was historically classified as poorly differentiated or undifferentiated adenocarcinoma.^5)^ Medullary carcinoma predominantly occurs in older patients.^5)^ The characteristics of medullary carcinoma include a female predominance, with a predilection for the right colon. Previous studies have reported that approximately two-thirds of medullary carcinoma cases occur in patients with right-sided colon cancer.^8)^ Several studies have reported that most medullary carcinomas are T3 tumors without lymph node metastasis (stage IIA), and that these tumors tend to be larger, with a median tumor size of 7 cm.^8)^ In our case, the patient was an 88-year-old woman with a T3 tumor without lymph node metastasis, which is consistent with the typical presentation of this carcinoma.

Medullary carcinoma of the colon shows a sheet-like, solid growth pattern that histologically resembles poorly or undifferentiated adenocarcinoma. Nongranular, homogenous malignant cells with intraepithelial lymphocytic infiltration were the predominant histological findings.^6,9)^ Several studies have reported that CDX2 expression is often negative, whereas loss of MLH1 expression is frequently observed, reflecting mismatch repair deficiency (dMMR) and MSI-H status.^10)^ Therefore, although medullary or undifferentiated carcinoma is difficult to diagnose by HE staining alone, it can be diagnosed by immunohistochemical staining for MLH-1 or CDX2.^8)^ Moreover, cadherin 17 and SATB2 are highly sensitive and specific markers for medullary carcinoma, with positivity rates of approximately 90%.^11)^

In our case, a histological examination demonstrated eosinophilic cytoplasm, reticulate-to-dense foci, and indistinct polarity of the dense foci. HE staining revealed lymphocyte infiltration. Focal CDX2 and MUC2 positivity were identified by immunohistochemistry, findings that supported the diagnosis of medullary carcinoma. Additional immunohistochemical analysis demonstrated dMMR, characterized by loss of MLH1 and PMS2 expression with preserved expression of MSH2 and MSH6. Taken together, these characteristic histological and immunohistochemical findings strongly supported the diagnosis of medullary carcinoma.

There has been no clear consensus on 5-fluorouracil (5-FU) resistance or on the efficacy of oxaliplatin (L-OHP) in MSI-H colorectal cancer, and the benefits of adjuvant chemotherapy remain unclear.^12–14)^ However, adjuvant chemotherapy combining 5-FU and L-OHP has been shown to significantly improve disease-free survival (DFS) and OS in patients with stage III MSI-H colorectal cancer, suggesting its potential role as a standard adjuvant treatment.^15)^ Although this case had high-risk features for Stage II disease (poorly differentiated histology, Ly1b, V1b, and Pn1a), adjuvant chemotherapy was not administered, considering the patient’s advanced age and the relatively favorable prognosis associated with medullary carcinoma.

## CONCLUSIONS

In general, treatment decisions for colorectal cancer should be based primarily on tumor stage. This is particularly relevant in older patients, in whom less invasive strategies may be preferentially considered. Although this tendency may be more pronounced in cases of poorly differentiated or large tumors, medullary carcinoma is known to have a relatively favorable prognosis. Therefore, excessive therapeutic de-escalation should be avoided. While preoperative histological diagnosis is often difficult, awareness of the clinical features suggestive of medullary carcinoma may support consideration of standard curative resection when appropriate. In older patients, treatment decisions should be individualized through shared decision-making, taking into account the disease prognosis, functional status, frailty, life expectancy, treatment-related risks and benefits, and patient preferences.

## DECLARATIONS

### CARE Checklist (2016) statement

The authors have read the CARE Checklist (2013), and the manuscript was prepared and revised according to the CARE Checklist (2016). Informed consent statement: Written informed consent was obtained from the patient for publication of this case report and any accompanying images.

### Availability of data and materials

The datasets supporting the conclusions of this article are provided within the manuscript.
